# CD4 binding determinant mimicry for HIV vaccine design

**DOI:** 10.3389/fimmu.2012.00383

**Published:** 2012-12-17

**Authors:** Yasuhiro Nishiyama, Stephanie Planque, Carl V. Hanson, Richard J. Massey, Sudhir Paul

**Affiliations:** ^1^Chemical Immunology Research Center, Department of Pathology and Laboratory Medicine, University of Texas-Houston Medical SchoolHouston, TX, USA; ^2^Viral and Rickettsial Disease Laboratory, California Department of Public HealthRichmond, CA, USA; ^3^Covalent Bioscience, Inc.Houston, TX, USA

**Keywords:** B cell superantigen, catalytic antibody, CD4 binding determinant, human immunodeficiency virus, neutralizing antibody, vaccine

## Abstract

The immunodominant epitopes expressed by the HIV-1 envelope protein gp120 are hypermutable, defeating attempts to develop an effective HIV vaccine. Targeting the structurally conserved gp120 determinant that binds host CD4 receptors (CD4BD) and initiates infection is a more promising route to vaccination, but this has proved difficult because of the conformational flexibility of gp120 and immune evasion mechanisms used by the virus. Mimicking the outer CD4BD conformational epitopes is difficult because of their discontinuous nature. The CD4BD region composed of residues 421–433 (CD4BD^core^) is a linear epitope, but this region possesses B cell superantigenic character. While superantigen epitopes are vulnerable to a small subset of spontaneously produced neutralizing antibodies present in humans without infection (innate antibodies), their non-covalent binding to B cell receptors (BCRs) does not stimulate an effective adaptive response from B cells. Covalent binding at naturally occurring nucleophilic sites of the BCRs by an electrophilic gp120 (E-gp120) analog is a promising solution. E-gp120 induces the synthesis of neutralizing antibodies the CD4BD^core^. The highly energetic covalent reaction is hypothesized to convert the abortive superantigens–BCR interaction into a stimulatory signal, and the binding of a spatially distinct epitope at the traditional combining site of the BCRs may furnish a second stimulatory signal. Flexible synthetic peptides can detect pre-existing CD4BD^core^-specific neutralizing antibodies. However, induced-fit conformational transitions of the peptides dictated by the antibody combining site structure may induce the synthesis of non-neutralizing antibodies. Successful vaccine targeting of the CD4BD will require a sufficiently rigid immunogen that mimics the native epitope conformation and bypasses B cell checkpoints restricting synthesis of the neutralizing antibodies.

## Introduction

In 2008, an estimated 33.4 million people worldwide were living with HIV/AIDS; 2.7 million people were newly infected; and 2.0 million people died due to AIDS. Anti-retroviral therapy (ART) suppresses viral replication and increases life expectancy. However, ART does not eradicate the infection, resistant viruses emerge, and adverse effects are frequent. Also, ART is costly, and sustaining affordable ART coverage in resource-poor regions where HIV is endemic is difficult. Developing a safe and efficacious vaccine represents the best long-term solution to combating HIV.

No effective vaccine has emerged from over 170 clinical trials that evaluated more than 30 vaccine candidates for induction of neutralizing antibodies, cytotoxic T cells or both arms of the immune system (McElrath and Haynes, 2010; Munier et al., 2011; Koff, 2012). Neutralizing antibodies, which can block infection by the free virus, are crucial to protection. Cytotoxic T cells can lyse infected cells, but have no effect on free virions. The root cause is the genetic sequence variability of the antigenic epitopes of HIV created by its error-prone reverse transcriptase enzyme. Several genetically distinct Group M HIV-1 subtypes have been identified (A1, A2, B, C, D, F1, F2, G, H, J, K; recombinant forms CRF01–CRF54) (Los Alamos National Laboratory Theoretical Biology and Biophysics Group, 2012). Any protective immune response to the epitopes induced by the infection is transient, as the virus quickly develops resistance mutations, and the adaptive immune response is too slow to keep pace with the generation of the viral quasispecies. As anti-HIV antibodies and cytotoxic T cells usually recognize the mutable envelope regions, the infection is not controlled, and thousands of diverse HIV strains have evolved world-wide.

The recent RV144 trial tested a vaccine candidate composed of gp120 protein and a *gp120/gag/protease* expression vector (Rerks-Ngarm et al., 2009). This was the first trial showing a statistically significant reduction in the risk of contracting HIV infection, but the risk reduction was marginal, and the results are not clinically meaningful in slowing the pandemic. An envelope vaccine derived from a single HIV strain may be conceived to provide some protection against infection by strains that express sufficiently similar mutable epitopes, but it is practically impossible to design a single vaccine immunogen capable of inducing antibodies that are broadly reactive with the mutable antigenic epitopes expressed by genetically divergent HIV strains.

To develop a broadly protective vaccine, it is necessary to target an HIV determinant that is structurally constant across the range of Group M HIV-1 strains responsible for infection world-wide. Moreover, it is important that the target determinant fulfills an essential role in infection—otherwise antibodies to the determinant may not neutralize the virus. Many recent vaccine development efforts have been focused at the envelope determinants essential for interaction with host receptors. Such viral determinants are likely to be structurally conserved to maintain the ability to the virus to infect cells.

## Overview of the CD4BD

The envelope glycoprotein complex (Env) on the HIV surface mediates entry into host cells and represents a prime target for neutralizing antibodies (Pantophlet and Burton, 2006). Env consists of homotrimeric complexes of non-covalently associated gp120-gp41 heterodimers derived by proteolytic processing of the precursor gp160 protein. The surface required for CD4 receptor binding is composed of gp120 residues with no involvement from gp41. Initial HIV binding to host cell CD4 receptors is an obligatory step for infection by all HIV-1 subtypes. This provides a selective pressure for structural conservation of the gp120 determinant that binds CD4 (the CD4 binding determinant, CD4BD), and the CD4BD has remained mostly conserved while the sequence of other gp120 regions has increasingly diverged as various Group M HIV-1 strains have evolved over the course of the pandemic in the last 3 decades.

**Figure 1:**
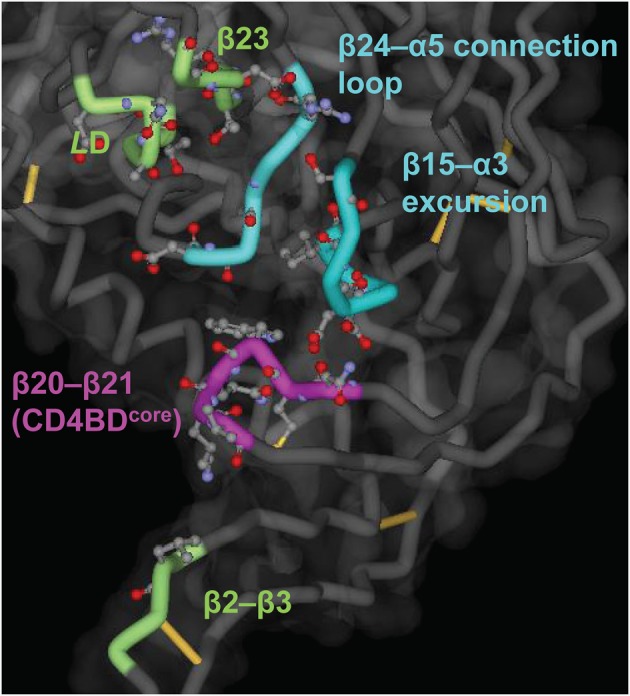
**Putative CD4BD structure.** The structure was deduced from the complex of gp120 (core), soluble CD4 and antibody 17b (Kwong et al., 1998). The main chain is shown as a tube, with the CD4BD^OD^ elements of the so-called Phe43 pocket in blue (the cavity into which CD4 residue Phe43 penetrates) and the CD4BD^core^ elements located in the bridging sheet in purple. Other CD4 contacting residues are in green. Side chain atoms of CD4 contacting residues are shown as ball-and-stick structures. Created with PDB data 1GC1.

The approximate spatial localization of the CD4BD has been deduced from crystallography of gp120 that was experimentally “rigidified” by excision of certain flexible segments (Kwong et al., 1998) together with site-directed mutagenesis followed by analysis of CD4 binding and monitoring of infectivity (Olshevsky et al., 1990). The complete CD4BD is a discontinuous determinant composed of residues distributed over six structural segments of gp120 (Figure 1): (1) the V1/V2 stem (β2–β3 segments), (2) loop *L*D, (3) the β15–α3 excursion from the gp120 surface, (4) the β20–β21 hairpin, (5) the β23 strand, and (6) the β24–α5 connection loop. All but the β2–β3 and β20–β21 segments of the CD4BD are located in the so-called gp120 “outer” domain, a designation meant to imply that this gp120 region is not proximal to the gp41 membrane anchor (Kwong et al., 1998). In the unliganded state of SIV gp120, the β2–β3 and β20–β21 hairpin regions are separated away from each other by about 20–25Å (Chen et al., 2005). Unliganded HIV gp120 structures are available, but these structures contain deletions that simulate the CD4-bound state of the protein (Kwon et al., 2012). The CD4BD portion located in the outer domain (CD4BD^OD^) is suggested to mediate initial weak contacts with CD4 (Zhou et al., 2007). This interaction is proposed to trigger a conformational rearrangement that translocates the β2–β3 and β20–β21 hairpins toward each other, forming the bridging sheet between the outer and “inner” domains.

Eight CD4 contacting amino acid residues are clustered in the linear stretch of amino acids 421–433 located in the β20–β21 bridging sheet region, corresponding to 33% of the overall CD4BD (Kwong et al., 1998). This includes the contiguous 425–430 region. Many of these residues are essential for virus infection as shown by mutagenesis studies (Lasky et al., 1987; Cordonnier et al., 1989). Consequently, the 421–433 region constitutes the critical core of the CD4BD (CD4BD^core^). If the CD4 binding model entailing initial, weak binding at the CD4BD^OD^ from crystallography studies is correct (Zhou et al., 2007), at a minimum CD4 contacts at the CD4BD^core^ are essential for subsequently strengthened, high affinity CD4-HIV Env binding needed to express the binding site for the co-receptor (for most primary isolates, CC-chemokine receptor type 5). As noted in the next section, synthetic CDBD^core^ peptides bind CD4 with modest affinity with no dependence on the remaining components of the overall CD4BD, and we do not exclude a requirement for CD4BD^core^ at the earliest CD4-HIV interaction stages.

## Potential of CD4BD^OD^ and CD4BD^core^ as HIV vaccine targets

The search for model neutralizing antibodies to the CD4BD that neutralize genetically divergent HIV strains and CD4BD-based immunogens that can induce the synthesis of such neutralizing antibodies has been ongoing for over two decades. As CD4 binding initiates infection, an antibody that binds an essential epitope within the CD4BD is predicted to neutralize the virus by a steric hindrance mechanism. In addition, a class of antibodies that catalyze the hydrolysis of gp120 following initial non-covalent binding to the CD4BD with HIV neutralizing activity was described by our group (Paul et al., 2004; Planque et al., 2007). Other “non-neutralizing” mechanisms of viral inhibition are also feasible. For example, antibodies may facilitate removal of virus-infected cells by natural killer cells. This mechanism is designated antibody-dependent cell-mediated viral inhibition, involving recognition of an HIV epitope by the antibody variable domains, followed by binding of the antibody constant domain region to Fc receptors expressed by natural killer cells (Bialuk et al., 2011).

The CD4BD, however, is poorly immunogenic, and difficulties were encountered in identifying neutralizing anti-CD4BD antibodies. In HIV primary isolates, virtually the entire outer domain surface is covered by glycans, and only the CD4-binding loop and neighboring surfaces are solvent exposed. Sugars are generally poorly immunogenic, and the glycan “shield” is thought to reduce the antibody response to the shielded surface (Wyatt et al., 1998; Wei et al., 2003). However, the exposed segments of the CD4BD also fail to induce an adequate antibody response (see next section). Other mechanisms underlying the poor immunogenicity of the CD4BD^OD^ and CD4BD^core^ must be identified and bypassed to design an HIV vaccine.

Selection of a target epitope within the CD4BD is key step in designing a vaccine that can induce a broadly neutralizing antibody response (Table 1). Discussed below are considerations likely to influence the neutralizing efficacy of various antibodies to the CD4BD and induction of their synthesis.

**Table 1:** **gp120 CD4BD neutralizing epitopes**.

**Antibody epitope**	**Structure, location**	**Neutralizing antibodies**	**Antibody source**	**Neutralization properties**
CD4BD^OD^	Discontinuous amino acid residues including CD4 contacting residues in the outer domain	IgG b12	Infected human (presumed subtype B virus)	Broad and potent inhibition of pseudovirus entry into cells engineered to display receptors for HIV; For some antibodies, neutralization of PBMC infection by HIV primary strains is unknown; Appearance of resistant viruses
		IgG VRC01	Infected human (subtype B virus)	
		IgG NIH45–46	Same donor as VRC01	
		IgG PGV04	Infected human (subtype A/D hybrid virus)	
CD4BD^core^	Linear epitope consisting of residues 421–433; Antibodies YZ23 and 3A5 also recognize a conserved V3 stem	IgG YZ23	E-gp120-immunized mice	Broad and potent neutralization of PBMC infection of HIV primary isolates; No inhibition of pseudovirus entry into engineered cells, suggesting distinct CD4BD^core^ dependence of infection in two assay systems
		IgG 3A5	E-gp120-immunized mice	
		scFv JL413	Non-infected human (lupus patients)	
		scFv JL427	Same donor as JL413	
		IgA LTS	Polyclonal serum IgA from long-term survivors of HIV infection (19–21 years)	

### CD4BD^OD^

Antibodies to the CD4BD^OD^ were cloned from HIV infected patients. Most anti-CD4BD^OD^ antibodies did not neutralize genetically diverse primary HIV isolates (Herrera et al., 2003; Binley et al., 2004). For over decade, the sole exception was the monoclonal antibody b12 with comparatively broad neutralizing activity directed to genetically heterologous HIV strains (Burton et al., 1994). Antibody b12 neutralized about 40% of the multi-clade pseudovirus panel tested (Binley et al., 2004; Walker et al., 2009; Corti et al., 2010). Neutralization potency for infection of the host cells engineered to express the type 5 chemokine receptor by pseudovirus strains was often superior to infection of human lymphocytes by primary HIV isolates (Binley et al., 2004). Intravenous infusion of antibody b12 protected rhesus macaque monkeys against vaginal challenge with simian human immunodeficiency virus engineered to express the HIV gp120 gene (Parren et al., 2001). The antibody also protected immunodeficient mice populated with human lymphocytes and then challenged with HIV (Gauduin et al., 1997). With the advent of improved B cell cloning technologies, several additional CD4BD^OD^ antibodies with broad and potent neutralizing activity have been identified recently. Among the broadest and most potent monoclonal antibodies of this class are VRC01, NIH45-46, and PGV04 (90% of pseudovirus strains tested were neutralized) (Wu et al., 2010; Scheid et al., 2011; Falkowska et al., 2012). The protective efficacy of antibodies to the CD4BD^OD^ in humans has not been tested.

Structural analysis of neutralizing and non-neutralizing antibodies to the CD4BD^OD^ has highlighted the difficulty in designing an immunogen that can induce the synthesis of the neutralizing antibody variety. The neutralizing and non-neutralizing anti-CD4BD^OD^ antibodies typically bind monomeric gp120 with comparable binding affinity and their binding determinants are largely common (Moore and Sodroski, 1996; Pantophlet et al., 2003; Zhou et al., 2007, 2010; Wu et al., 2011). Yet the non-neutralizing antibodies showed little or no binding to the Env expressed on cell-surface (which was assumed to mimic the functional gp120 structure on the virus surface) (Pancera and Wyatt, 2005). Clearly, monomer gp120 binding data cannot be extrapolated to recognition of the native CDBD^OD^. CD4-gp120 is thought to trigger an allosteric conformational change that exposes the coreceptor binding site. Antibodies to the CD4BD^OD^ with and without neutralizing activity induce distinct conformational changes in monomer gp120 (Zhou et al., 2007; Chen et al., 2009; Falkowska et al., 2012), but these analyses have not provided guidance in rational design of an immunogen that can induce the synthesis of the neutralizing antibodies.

If the role of the CD4BD^OD^ in CD4 binding is limited to providing initial weak contacts, an antibody that mimics exactly the mechanism of CD4-CD4BD^OD^ binding may not be sufficient to interfere with the autonomous binding interactions of CD4 with the remaining CD4BD components. For more effective neutralization, therefore, it is desirable to induce antibodies that make additional high affinity contacts with the remaining CD4BD components or spatially remote gp120 residues that control the CD4BD conformation. In the case of antibody VRC01, unique contacts not directly involved in CD4 binding include certain sugars in the outer domain and amino acids in the inner domain region (Zhou et al., 2010). However, an antibody specificity outside the functionally essential CD4BD also increases the likelihood that antibody-resistant virus strains will emerge. Indeed, while antibody VRC01 neutralizes heterologous virus strains, the dominant autologous HIV quasispecies in the patient from whom the antibody was isolated were antibody-resistant (Wu et al., 2012). Promising attempts to improve the epitope specificity of antibodies to the CD4BD^OD^ by a rational, laboratory-level mutagenesis approach have been reported. Introducing a mutation in such an antibody designed to increase contact with the gp120 β21 sheet region that helps form the CD4BD^core^ epitope resulted in increased neutralization potency and breadth (Diskin et al., 2011), supporting the potential value of an immunogen that induces antibody responses to gp120 regions outside the CD4BD^OD^.

**Figure 2:**
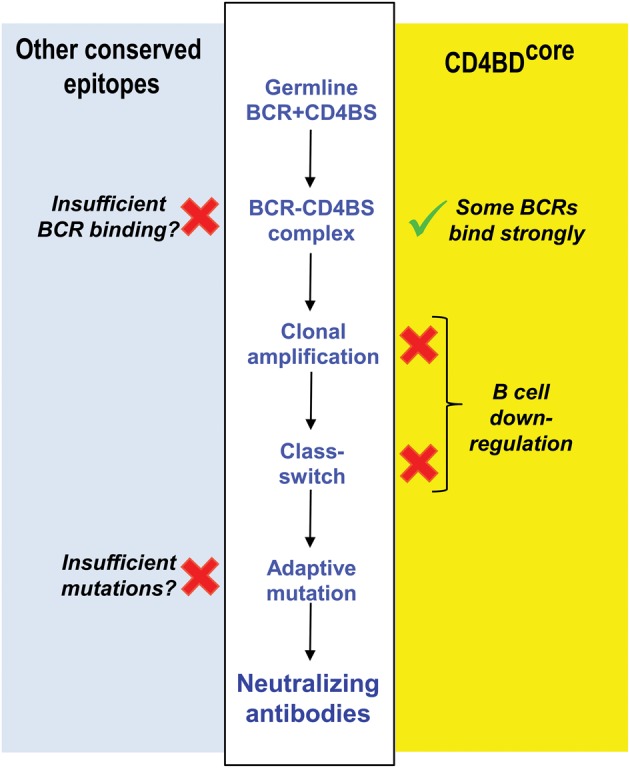
**Reasons underlying failed induction of antibodies to CD4BD.** Competing hypotheses advanced to explain failure of adaptive antibody synthesis to the CD4BD. *Middle white panel:* regulatory check-points in B cell differentiation influencing neutralizing antibody production. *Right yellow panel:* the CD4BD^core^ is a B cell superantigenic epitope that binds the FRs of constitutive BCRs non-covalently, but this causes B cell down-regulation. In contrast, covalent vaccination up-regulates B cells. *Left light blue panel:* other popular hypotheses based on studies by Dimitrov and coworkers, Scripps Institute investigators and NIH Vaccine Research Center investigators. Sufficient V gene mutations needed to bind CD4BD do not occur. Alternatively, the affinity of conserved HIV epitopes for BCRs is too low. NAbs, neutralizing antibodies. X, weakness in B cell response. √, our proposed basis for HIV vaccination.

The difficulties in isolating broadly neutralizing antibodies to the CD4BD^OD^ and understanding their behavior have inspired conflicting hypotheses invoking intrinsic weaknesses in the humoral immune system as the cause, that is, an inability of germline antibodies to recognize the CD4BD^OD^ vs. a failure of the adaptive phase of the antibody response. Inducing antibody synthesis involves initial weak immunogen binding to B cell receptors (BCRs) with V regions encoded by germline genes (BCRs; antibodies associated with signal transducing proteins), followed by immunogen-driven clonal expansion of B cells and selection of BCRs containing mutated complementarity determining regions (CDRs) expressing improved antigen binding affinity. The BCR repertoire expressed constitutively prior to contact with immunogen is diverse and large, composed of paired light chain and heavy chain variable domains (V_L_ and V_H_ domains) generated from about 500 V, D, and J germline genes by V-(D)-J gene recombination, a step that introduces sequence diversity in CDR3 (Janeway et al., 2005). The diversity of constitutive BCRs is innate in the sense that it is generated randomly with no requirement for contact with immunogen. Dimitrov and coworkers suggested that an unusually low binding affinity of conserved HIV epitopes for constitutive BCRs precludes B cell recruitment into the adaptive differentiation pathway (Xiao et al., 2009) (Figure 2). Conversely, the neutralizing antibody VRC01 cited in the preceding paragraph contains extremely dense V region somatic mutations (Scheid et al., 2011; Wu et al., 2011), which prompted the hypothesis that B cells are incompetent in generating antibodies containing a sufficient number of adaptive mutations upon exposure to HIV. The VRC-like antibodies originate exclusively from the VH-1 antibody family containing the germline-encoded VH Arg71 residue important for CD4BD^OD^ recognition (West et al., 2012), suggesting the importance of pre-existing germline antibody repertoire in mounting an adaptive antibody response to the CD4BD^OD^.

### CD4BD^core^

**Figure 3:**
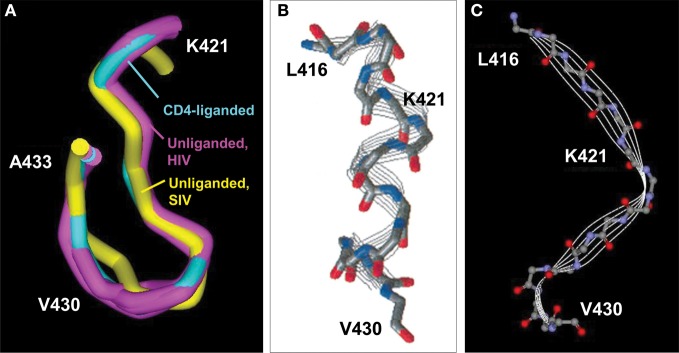
**CD4BD^core^ conformations. (A)** Superimposed CD4BD^core^ conformations in gp120 variants (residues 421–433) complexed with soluble CD4 and antibody 17b (cyan; 1GC1) and gp120 (extended core) without a ligand (purple; 3TGQ, 3TGR, 3TGT, 3TIH). For comparison, the CD4BD^core^ of unliganded SIV gp120 is included (yellow; 2BF1). Protein backbones were superimposed onto the CD4-bound structure. **(B)** Structure of a synthetic peptide corresponding to gp120 416–430 determined by nuclear magnetic resonance spectroscopy (NMR) in 2,2,2-trifluoroethanol. **(C)** Structure of the corresponding 416–430 region in the gp120 protein determined by X ray crystallography (1GC1). The NMR-derived peptide structure is reprinted with permission from reference Mihailescu et al. (2002) (Copyright 2002 American Chemical Society).

The CD4BD^core^ is structurally distinct from the CD4BD^OD^. Except in the early years of HIV vaccine research, dominant research groups in the field have held that the CD4BD^OD^ is the best target epitope for vaccination, and little attention was paid to the CD4BD^core^ as an alternative worthy of detailed structural discussion. However, the pdb files of various crystal structures of “rigidified” monomer gp120 contain important structural details that are consistent with the suitability of the CD4BD^core^ as a vaccine target (*e.g*., CD4 liganded 1GC1, 1G9M, 1G9N, 1RZJ, 1RZK, 3JWD, 3JWO, 2NXY, 2NXZ, 2NY5, 2B4C, 2QAD; unliganded 2BF1, 3TGQ, 3TGR, 3TGT, 3TIH; CD4BD^OD^ antibody liganded 3NGB, 3SE8, 3SE9, 3U7Y, 2NY7, 3HI1, 3IDX; low-molecular-weight CD4 mimetic liganded 1YYL, 1YYM, 2I5Y, 2I60, 3TGS). The solvent-exposed area of the CD4BD^core^ (residues 421–433) is 971–1023 Å^2^ (Kwong et al., 1998, 2000; Chen et al., 2005; Huang et al., 2005), exceeding the area needed for high affinity antibody binding (Wilson et al., 1991). While translational movement of the CD4BD^core^-containing bridging sheet upon CD4 binding has been emphasized (Kwong et al., 2002), the crystal structures provide no support for the occurrence of a conformational change within the CD4BD^core^ (Figure 3A). There is no evidence, therefore, that the CD4-binding function of the CD4BD^core^ is sterically unfavorable or depends on a temporally preceding allosteric conformational transition.

Linear synthetic peptides encompassing the CD4BD^core^ are conformationally flexible in aqueous solution. Circular dichroism and nuclear magnetic resonance studies failed to identify a preferred conformation unless solvents providing a hydrophobic environment are included (Graf Von Stosch et al., 1995). Interestingly, in such solvents, the CD4BD^core^ synthetic peptides assume a helical conformation that binds CD4 autonomously with moderate binding affinity (Reed and Kinzel, 1991; Robey et al., 1996). Inclusion of the N terminal pentapeptide L-P-C-R-I corresponding to residues 416–420 also favors assumption of a helical conformation by the CD4BD^core^ residues 421–433 with improved CD4-binding capability (Reed and Kinzel, 1993). The solution-state helical CD4-binding conformation differs from the β-fold CD4BD^core^ state identified in the model monomer gp120 crystals (Figures 3B,C), and it is not clear whether the crystal structures are useful models for the unliganded and CD4-bound states of the native trimeric gp120 on the HIV surface.

B cells and the CD4BD^core^ have a remarkable relationship. The CD4BD^core^ overlaps the B cell superantigen site of gp120. Like other B cell superantigens, gp120 is recognized non-covalently by the framework regions (FRs) of pre-immune antibodies produced without prior HIV exposure (constitutive antibodies) (Townsley-Fuchs et al., 1997; Silverman and Goodyear, 2006). Several groups reported that antibodies produced spontaneously by humans without HIV infection or vaccination bind the CD4BD^core^-spanning superantigenic determinant of gp120 and synthetic peptides containing the CD4BD^core^ (Berberian et al., 1993; Goodglick et al., 1995; Karray and Zouali, 1997; Karray et al., 1998; Neshat et al., 2000). Superantigen epitopes are recognized by antibodies containing V regions in the germline configuration with no requirement for an immunogen-driven antibody response (Silverman and Goodyear, 2006). Rare CD4BD^core^-specific antibody fragments that neutralize diverse HIV strains potently have been isolated from phage display libraries prepared from non-infected humans (single chain Fv fragments JL413, JL427) (Karle et al., 2004; Planque et al., 2012b). The binding occurs mostly at germline gene-encoded structural elements in the FRs and certain CDR residues outside the traditional antigen-binding pocket formed by the CDRs (Berberian et al., 1993; Karray et al., 1998; Neshat et al., 2000).

Braun's group studied the relationship between the levels of reversibly binding IgGs to the gp120 superantigen site in non-infected humans and the risk of contracting HIV infection (Townsley-Fuchs et al., 1996). Increased binding was correlated with reduced incidence of HIV infection. Like the innate reversible binding activity for superantigens, some antibodies express a germline V gene encoded catalytic activity (Gao et al., 1995; Gololobov et al., 1999; Reshetnyak et al., 2007; Sharma et al., 2009; Hifumi et al., 2012). Upon completion of the non-covalent CD4BD^core^ binding step, a subset of the constitutive antibodies was reported to catalyze the hydrolysis of gp120 by a nucleophilic, serine protease mechanism (Paul et al., 2004; Planque et al., 2007). The catalytic property is expressed at greatest levels by antibodies of the IgM and IgA classes, and the secretory IgA from non-infected humans neutralized HIV (Planque et al., 2007). Importantly, the antibodies from various non-infected humans recognize the CD4BD^core^ at highly variable levels, and only a small subset of the innate antibodies neutralize HIV with potency that is sufficiently great to anticipate protection against the infection (Planque et al., 2012b).

The reversible binding and catalytic properties of the pre-existing antibodies may furnish limited innate protection against HIV (Townsley-Fuchs et al., 1996; Planque et al., 2007, 2012b). There is, however, a heavy cost—the failure of adaptive B cell immunity. Stimulatory antigen binding at antibody CDRs drives B cell clonal selection. By contrast, superantigen binding at the FRs likely induces a distinct cellular signaling pathway(s) that causes apoptosis, thereby counteracting any stimulatory B cells signals needed to develop a mature class-switched antibody response (Silverman and Goodyear, 2006; Goodyear et al., 2007). Superantigen-FR binding, therefore, may be viewed as down-regulatory event (Figure 2). Infected humans and animals immunized with gp120 rarely generate class-switched neutralizing antibodies to the CD4BD^core^ by adaptive immune mechanism (Sun et al., 1989; Kelker et al., 2010). It may be hypothesized that vulnerable epitopes essential for microbial survival have evolved superantigenic character as an immune evasion mechanism that precludes an efficient adaptive antibody response (Goodyear and Silverman, 2005; Planque et al., 2008). Rapid induction of B cell adaptive immunity is a stochastic process driven by certain high probability cellular signaling pathways. Adaptive synthesis of the antibodies by CD4BD^core^ binding at the BCR FRs, on the other hand, is a low probability event. The prohibition on producing neutralizing antibodies to the CD4BD^core^ does not appear to be an absolute one. We reported CD4BD^core^-specific antibodies with potent neutralizing activity directed to genetically heterologous HIV strains in the blood of three hemophilia A patients who survived infection for over two decades with little or no requirement for ART (Planque et al., 2010). It may be hypothesized that upon sufficiently prolonged stimulation by HIV, the immune system can bypass the restriction on adaptive synthesis of neutralizing antibodies to the CD4BD^core^ epitope. The three hemophilia A patients were coinfected with hepatitis C virus and had received extensive Factor VIII replacement therapy. Consequently, further studies will be needed to rule out fortuitous immune stimulatory events unique to these patients as the cause of CD4BD^core^-specific antibody production.

**Figure 4:**
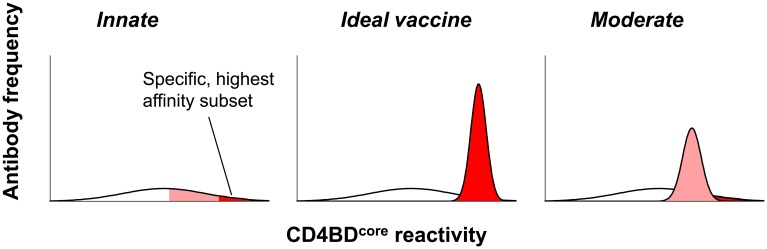
**Constitutive CD4BD^core^-reactive antibodies pertinent to HIV vaccination.** *Left panel:* model of innate germline antibody repertoire containing a wide range of antibodies with varying CD4BD^core^-reactivity and HIV neutralizing potency. A small antibody subset with the greatest CD4BD^core^-reactivity is hypothesized to possess potent and broad neutralizing activity (red). *Middle panel:* an electrophilic vaccine expressing a CD4BD^core^ that mimics the viral CD4BD^core^ conformation accurately will selectively amplify the broadly neutralizing antibody subset with greatest CD4BD^core^-reactivity. *Right panel:* a vaccine that mimics the viral CD4BD^core^ imperfectly will amplify an antibody subset with lesser CD4BD^core^-reactivity and neutralizing activity (pink).

**Figure 5:**
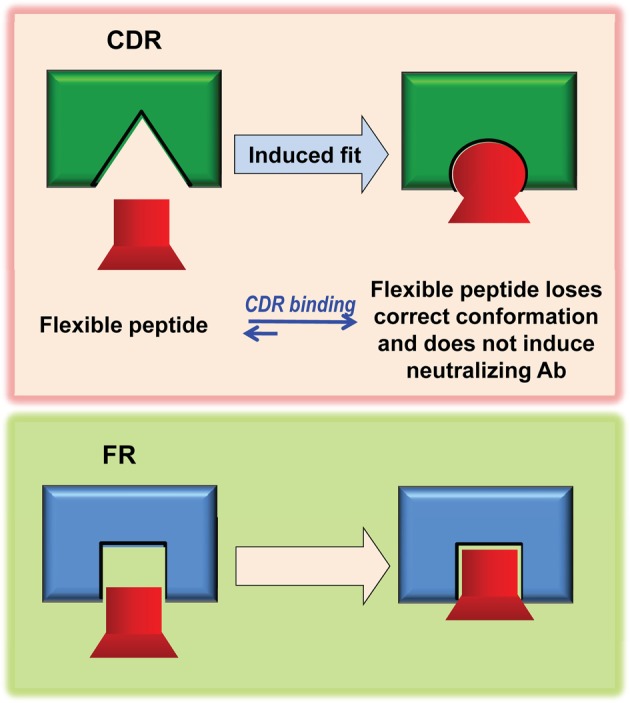
**Hypothetical mechanism for induction of non-neutralizing antibodies by flexible CD4BD^core^ mimetics.** A flexible mimetic can detect innate antibodies (or B cell receptors) that utilize the FRs for CD4BD^core^ recognition and induce the synthesis of such antibodies if mechanisms for productive B cell stimulation can be devised (*Bottom*). When used as an immunogen, the conformation of the flexible mimetic can adapt to the structure of the B cell receptor CDRs by the induced-fit mechanism, which will corrupt the adaptive antibody response and skewed the response toward production of non-neutralizing antibodies directed to the incorrect CD4BD^core^ conformation (*Top*).

As innate, FR-based CD4BD^core^-specific antibodies neutralize HIV, rapid amplification and improvement of these antibodies may serve as a viable basis for developing an HIV vaccine (Figure 4). Success, however, will require solutions to these broad problems. First, the physiological restriction on vaccine-driven synthesis of antibodies dependent on their FRs for HIV neutralization must be overcome. Second, CD4BD^core^ conformational flexibility must be controlled. Synthetic peptides were useful in discovering the innate, FR-dependent antibodies to the CD4BD^core^. Small peptides, however, are flexible, and they assume alternate conformations depending on their microenvironment. The importance of controlling CD4BD^core^ conformation is evident from reports of vigorous production of antibodies to synthetic peptides that displayed inconsistent HIV neutralizing activity and varying binding specificity (Neurath et al., 1990; Morrow et al., 1992; Karle et al., 2003). While the CD4BD^core^-spanning peptides detect pre-existing neutralizing antibodies, inducing the synthesis of such antibodies is more onerous. If the dose of the CD4BD^core^ peptide immunogen is sufficient to saturate the FR-based binding sites, the peptide can also induce a classical adaptive antibody response by binding at the lower affinity CDR-based binding sites of germline BCRs (Figure 5). Peptide binding at the FRs will not induce antibody synthesis, as this interaction is thought to induce down-regulatory B cell signaling. Binding at the CDRs induces antibody synthesis, but antibody response may be dominated by irrelevant, non-neutralizing antibodies due to the induced-fit binding mechanism (Figure 5). Indeed, synthetic peptide immunogens often fail to induce the synthesis of neutralizing antibodies to the native conformation of the corresponding epitope in full proteins for the same reason, *i.e*., folding of peptides into irrelevant conformations induced by fitting into the germline antibody binding site (Leder et al., 1995; Wedemayer et al., 1997).

The conformation of peptide epitopes within the backbone of large proteins is usually more rigid and less sensitive to transitions upon microenvironmental perturbations compared to the synthetic peptides. Full-length, monomeric gp120 expresses the CD4BD^core^ in a conformation reactive with the innate, FR-dependent neutralizing antibodies (Nishiyama et al., 2009; Planque et al., 2010). Despite the evident existence of a conformationally correct, solvent-exposed CD4BD^core^ on the surface of gp120 (Nishiyama et al., 2009; Planque et al., 2010), immunization with this protein does not induce a robust adaptive anti-CD4BD^core^ antibody response (Sun et al., 1989; Kelker et al., 2010), and these antibodies are also poorly represented in the antibody response to HIV infection (Planque et al., 2010). The down-regulatory interactions at the BCR FRs, therefore, may be the primary factor limiting the CD4BD^core^-directed neutralizing antibody response to the monomeric gp120 immunogen and oligomeric gp120 on the HIV surface. As the CD4BD^core^ is recognized preferentially by the innate FRs compared to the CDRs, no induction of the CDR-based antibody response to this epitope is anticipated unless the monomer gp120 immunogen and infectious virions are presented to the immune system at an excess concentration.

## CD4BD mimetics as candidate vaccines

There is no doubt that HIV has evolved highly specialized mechanisms that enable its vulnerable epitopes to evade physiological immune responses. Previously tested candidate vaccines do not induce sufficient neutralizing antibodies to the CD4BD for prevention or control of the infection. This section describes attempts to engineer Env-based immunogens that might address the challenges in inducing the antibodies to CD4BD^OD^ and CD4BD^core^.

### CD4BD^OD^

It was suggested that the poor antibody response to the conserved CD4BD^OD^ epitopes was due to the superior intrinsic immunogenicity of gp120 variable domain epitopes (Boudet et al., 1992; Montefiori et al., 1993) and conformational flexibility of gp120 (Kwong et al., 2002). Conformational differences between the CD4BD^OD^ epitopes expressed by monomer gp120 and oligomeric gp120 on the viral surfaces have also been invoked to explain the poor CD4BD^OD^ antibody response induced by the former immunogen (Chen et al., 2009). Extensive and sophisticated projects were designed to develop analogs of gp120 that overcome these weaknesses, but no immunogen capable of inducing broadly neutralizing antibodies has emerged. For example, a mutant gp120 was prepared (PF2-gp120) by deleting the immunodominant V1, V2, and V3 loops and the N- and C-termini, and mutations were introduced (T257S and S375W) to mimic the CD4-bound state (Xiang et al., 2002). Oligomeric PF2-gp140 containing the GCN4 trimerization motif was prepared to replicate the trimeric Env spikes on the HIV surface (Dey et al., 2007). These analogs displayed limited selectivity in discriminating between binding to reference neutralizing and non-neutralizing antibodies to CD4BD^OD^ epitopes, but they did not induce the synthesis of broadly neutralizing antibodies in rabbits (Dey et al., 2007; Feng et al., 2012).

CD4BD^OD^ epitopes are composed of discontinuous gp120 regions composed of small peptide units and individual amino acids. Protein epitopes can adopt a multitude of conformations by the induced-fit mechanism along the binding reaction coordinate. Consequently, it is difficult to rationally design mimetics that replicate the functional CD4BD^OD^ epitope conformations. Peptides that mimic the CD4BD^OD^ have been sought based on binding of the antibody b12 from a random peptide library (Zwick et al., 2001). A 15-residue disulfide-linked dimer that bound the antibody with a μM K_D_ was identified. The peptide is homologous in sequence to gp120 loop *L*D, a CD4BD component. However, the antibody makes distinct types of contacts with the peptide versus gp120 according to crystallography, and the peptide mimics at most 1–2 binding sites of the antibody-reactive CD4BD^OD^ epitope (Saphire et al., 2007). Immunization with the peptide did not elicit neutralizing or gp120-binding antibodies (Saphire et al., 2007). Given the varying neutralizing properties of anti-CD4BD^OD^ antibodies with largely shared epitope specificity, it is hard to see how the peptide binding strength can be applied to develop a CD4BD^OD^ mimotope capable of inducing neutralizing antibody responses.

### CD4BD^core^

Like the hypothetical CD4BD^OD^-based vaccine, developing a CDBD^core^ immunogen that induces the synthesis of broadly neutralizing antibodies must solve basic science hurdles, chiefly, the problem of B cell superantigenicity. A potential solution to this problem has emerged serendipitously. Conformational mimicry of the native CD4BD^core^ is also essential. On the positive side, the CD4BD^core^ is a linear epitope. Synthetic CD4BD^core^ peptides are a useful starting point for identification of a probe that can accurately detect neutralizing antibodies, and potentially might induce the synthesis of neutralizing antibodies.

#### Electrophilic gp120 (E-gp120) analog

E-gp120 displays novel molecular properties that support its potential as a vaccine candidate capable of inducing neutralizing antibodies to the CD4BD^core^. It consists of glycosylated monomeric gp120 conjugated at Lys residues to electrophilic phosphonates via a linker (Paul et al., 2003). E-gp120 displayed soluble CD4 (sCD4) binding comparable to the non-electrophilic gp120, indicating a preserved CD4BD conformation. Several antibodies with neutralizing activity attributable to CD4BD^core^ recognition were bound by E-gp120 at levels somewhat superior to non-electrophilic gp120, indicating that the CD4BD^core^ epitope is accessible on the E-gp120 surface (the innately produced single chain Fv JL427, polyclonal IgA from survivors of prolonged HIV infection) (Nishiyama et al., 2009; Planque et al., 2010, 2012b). Binding of the anti-V3 monoclonal antibodies 447-52D and 268-DIV by E-gp120 was reduced compared to non-electrophilic gp120, suggesting selectively reduced accessibility or partial destruction of the gp120 V3 epitopes deriving from structural perturbations attendant to electrophile insertion (Nishiyama et al., 2009). Interestingly, E-gp120 spontaneously formed covalently bonded oligomers by virtue of an intermolecular reaction between the phosphonate electrophile and a naturally occurring gp120 nucleophilic site (Paul et al., 2003; Nishiyama et al., 2005, 2009). Similar nucleophilic sites serve as the catalytic sites of enzymes, and such sites were originally assumed to exist only in catalytic biomolecules. However, studies with small molecule electrophilic probes revealed the frequent distribution of the naturally occurring nucleophilic sites in non-catalytic proteins, including the non-catalytic gp120 protein and non-catalytic antibodies (Planque et al., 2003; Nishiyama et al., 2005).

**Figure 6:**
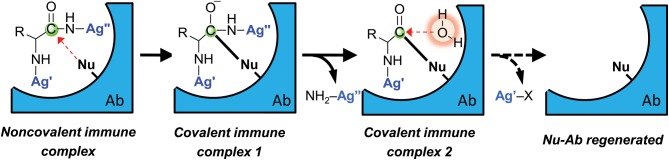
**Schematic representation of irreversible antigen (Ag) binding and catalytic Ag hydrolysis by a nucleophilic antibody (Ab).** Specificity is derived from non-covalent epitope–paratope binding. The antibody nucleophile (Nu) attacks the weakly electrophilic peptide bond carbonyl. Covalent immune complex 1 is a resonant stable complex formed prior to expulsion of C-terminal antigen fragment (NH_2_-Ag″). Covalent immune complex 2 is an acyl-antibody complex that is hydrolyzed to release the N-terminal antigen fragment (Ag′-X; X is an amino acid) when activated water is available in the antibody combining site. Ag′ and Ag″ are components of the epitope recognized by the antibody.

Like the non-covalent CD4BD^core^ binding capability, nucleophilic reactivity of antibodies is an innate property encoded by the germline V region genes (Planque et al., 2003). The E-gp120 immunogen was designed to stimulate the synthesis of antibodies with adaptively strengthened nucleophilic reactivity coordinated with improved non-covalent epitope recognition (Paul et al., 2003). We hypothesized that antibodies with strengthened nucleophilic reactivity would produce these outcomes (Figure 6): (1) Irreversible gp120 binding if the antibody active site does not support water attack on the covalent acyl-antibody complex; and (2) Catalytic gp120 hydrolysis if the water attack step in the hydrolytic reaction pathway is feasible. Both outcomes were observed. Monoclonal IgG antibodies to E-gp120 displayed irreversible gp120 and HIV binding (Nishiyama et al., 2006). Some of the IgGs catalyzed the hydrolysis of gp120 slowly (Paul et al., 2003). The limitation in catalytic rate derives from the IgG class constant domain scaffold—recent studies have shown that the same V domains of an innately produced anti-CD4BD^core^ antibody displayed far superior catalytic activity when expressed within the IgM scaffold compared to the IgG scaffold (Sapparapu et al., 2012), and polyclonal IgM preparations also displayed superior catalysis compared to IgG preparations from the same sera (Paul et al., 2004). The IgGs display no deficiency in non-covalent recognition, and the subsequent nucleophilic attack step results in irreversible gp120 binding, a reaction that is functionally equivalent to an infinite binding affinity event (Nishiyama et al., 2006). Deficient support of subsequent steps in the catalytic cycle in the IgG scaffold, however, can limit the rate of hydrolysis.

**Figure 7:**
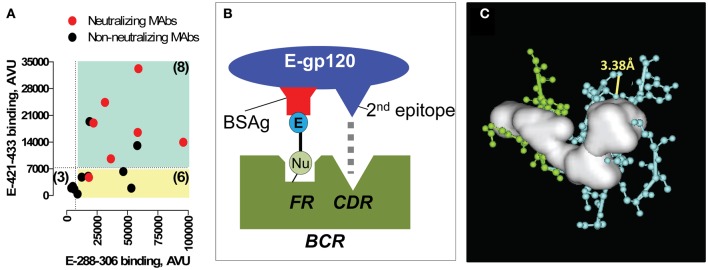
**Monoclonal antibodies (MAbs) raised to E-gp120. (A)** Epitope specificity-neutralizing activity relationships. Anti-E-gp120 MAbs frequently display binary E-288–306 and E-421–433 binding. Binding of E-288–306 and E-421–433 by 17 MAbs was measured. Plotted are band intensities (in arbitrary volume unit, AVU) of the adducts formed by individual MAbs. Cyan quadrant contains MAbs with adduct densities at least 4-fold greater than the background mean band intensity of adducts formed by the irrelevant control E-peptide probes (binary peptide binding MAbs). Yellow quadrant contains monoreactive MAbs to E-288–306. Red symbols denote MAbs that neutralized subtype C strain 97ZA009 (IC_50_ < 20 μg/mL); black symbols, non-neutralizing MAbs. Number in parentheses corresponds to the MAbs in the quadrant. **(B)** Binary epitope BCR-immunogen recognition model. E-gp120 binds BCR nucleophiles (Nu) covalently, releasing a large amount of energy that eliminates the physiological restriction on differentiation of innate CD4BD^core^-specific B cells. Concomitant CDR binding by the 2nd E-gp120 epitope also counteracts negative B cell signaling. **(C)** Expanded view of V_H_ FR-cavity fitted with the solid white object deduced by crystallography. Cyan, V_H_ FR residues; green, V_H_ CDR2 residues. Yellow line connects atoms that can form a nucleophilic diad by serving as the proton donor–acceptor pair (V_H_ T70 Oγ; V_H_ T70 carbonyl O). Panels **(A)** and **(C)** from reference Nishiyama et al. (2009).

Induction of neutralizing antibodies to the CD4BD^core^ by immunization with gp120 devoid of the electrophiles is a rare event (Sun et al., 1989; Kelker et al., 2010). Examination of epitope specificity using overlapping synthetic gp120 peptides yielded remarkable findings. First, monoclonal IgGs induced by the E-gp120 immunogen recognized the CD4BD^core^ frequently, an activity correlated with neutralization of genetically diverse HIV strains (Figure 7A) (Nishiyama et al., 2009). Second, the CD4BD^core^ recognition capability was accompanied by recognition of a second epitope that is spatially distant from the CD4BD^core^ in the gp120 crystal structure, an epitope located in the gp120 V3 stem. Crystallography and mutagenesis of an E-gp120 induced IgG (clone YZ23) suggested that binary epitope specificity is explained by CD4BD^core^ binding at the antibody FRs in concert with binding of the second epitope at the CDRs (Figures 7B,C) (Nishiyama et al., 2009). The replacement/silent mutation ratios for the antibody CDRs and V_H_ FRs of the monoclonal antibodies exceeded the ratios for a random mutational process, suggesting an immunogen-driven antibody synthetic response. We suggested these mechanisms underlying the neutralizing antibody induction: (1) Covalent binding of nucleophilic BCRs by E-gp120, a reaction generating a large amount of energy that can be used for stimulating productive B cell signalizing compared to the non-productive non-covalent binding of the CD4BD^core^ at the FRs; and (2) Stimulatory CDR-engagement by a second epitope occurring simultaneously with FR binding of the CD4BD^core^ epitope, which may compensate for down-regulatory signaling due to FR-CD4BD^core^ binding.

While further studies are needed to establish the immunological checkpoints restricting physiological synthesis of anti-CD4BD^core^ antibodies and the suggested mechanisms underlying the success of E-gp120, the antibody epitope specificity and neutralization studies indicate that the problem of CD4BD^core^ superantigenicity is solvable. The data indicate that insertion of electrophiles in gp120 converts the poorly immunogenic CD4BD^core^ into a strongly immunogenic neutralizing epitope. As the antibodies bind HIV, the CD4BD^core^ conformation expressed by E-gp120 must mimic the native epitope conformation on the HIV surface. It remains possible that E-gp120 induces the synthesis of antibodies to irrelevant CD4BD^core^ conformations in addition to antibodies to the correct epitope conformation found to neutralize HIV broadly. Effective vaccination will require that the latter antibody subset is represented sufficiently in the polyclonal antibody response induced by E-gp120. Ongoing analyses of macaque sera following immunization with E-gp120 have provided encouraging neutralization data about the quality of the polyclonal antibody response (Planque et al., 2012a).

#### E-416–433

The idea of focusing the antibody response at the CD4BD^core^ using a synthetic peptide immunogen is attractive, as this may eliminate irrelevant non-neutralizing antibodies to other epitopes and the offsetting effect of antibodies that might enhance HIV infection *via* the Fc receptor and complement receptor-dependent pathways (Takeda et al., 1988). There is no evidence that antibodies to the CD4BD^core^ enhance infection by such pathways. Unlike the CD4BD^OD^ epitope, the CD4BD^core^ contains contiguous amino acids necessary for CD4 binding. Linear synthetic peptides mimic the CD4BD^core^ conformation sufficiently to detect neutralizing antibodies. We employed probes residues 421–433 or 416–433 containing electrophilic phosphonate groups (E-421–433 and 416–433, respectively), to identify antibodies that neutralize primary HIV strains, including antibodies produced spontaneously by humans without HIV infection (Planque et al., 2007), patients with prolonged HIV infection (Planque et al., 2010) and animals immunized with E-gp120 (Nishiyama et al., 2009). Other groups employed non-electrophilic synthetic peptides to identify rare monoclonal antibodies to the CD4BD^core^ that neutralized laboratory-adapted HIV strains. The E-416–433 peptide binds CD4 better than E-421–433 (Nishiyama et al., 2009) on account of the helix stabilizing N-terminal Leu-Pro-Ser-Arg-Ile (Reed and Kinzel, 1993) and insertion of bulky side chain phosphonates may rigidify the peptides. However, the CD4 binding strength is still ~10-fold lower than E-gp120, presumably due to the absence of the CD4BD^OD^.

As noted in section “CD4BD^core^”, a peptide probe that detects neutralizing antibodies may not induce the synthesis of neutralizing antibodies. E-416–433 represents an improved mimetic of the CD4BD^core^, but a small, conformational flexible peptide can induce non-neutralizing antibodies to an irrelevant CD4BD^core^ conformation due to the induced-fit binding mechanism. Preliminary macaque studies suggest that E-gp120 may be the superior immunogen (Planque et al., 2012a). Factors in evaluating the immunogenicity of E-416–433 *versus* E-gp120 include the presence of the additional outer domain CD4 binding residues in the latter immunogen. The CD4BD^OD^ could potentially stabilize the CD4BD^core^ conformation, and E-gp120 could induce antibodies with overlapping CD4BD^core^/CD4BD^OD^ specificity. Both E-gp120 and E-416-433 contain electrophiles that bind BCRs covalently, but E-gp120 enjoys the advantage of binary docking of the CD4BD^core^ into the FR-based site and a second epitope into the CDR-based site. A single E-peptide molecule can only bind either at the FRs or the CDR, and CDRs that bind the incorrect CD4BD^core^ conformation may dominate the E-416–433 induced antibody response. Binary E-gp120-BCR docking, in contrast, facilitates production of Abs with the FR-based site that recognize the correct CD4BD^core^ conformation (Figure 5).

## Importance of HIV neutralization assays

Immunochemical measurements of antibody-CD4BD reactivity using purified gp120 or peptide probes do not predict faithfully the HIV neutralizing capability due to the problem of CD4BD conformational variability. Consequently, a central concern in vaccine development is valid measurement of HIV neutralizing activity using tissue culture assays. Antibodies to the CD4BD^core^ described in the preceding sections neutralize the infection of human peripheral blood mononuclear cells by genetically diverse strains found across the world (PBMC assay). This assay is often cited as the “gold standard” for determining HIV neutralization in tissue culture because it approximates the natural process of infection *in vivo*. However, discrepant neutralization by antibodies to various HIV epitopes in the primary virus-PBMC assay vs. the pseudovirion-engineered reporter cell assay were noted (Brown et al., 2008; Mann et al., 2009; Rusert et al., 2009; Paul et al., 2010; Heyndrickx et al., 2012), including the CD4BD^core^ epitope. The CD4BD^core^-specific antibodies produced by long-term survivors of HIV infection (Planque et al., 2010) or induced in mice by E-gp120 immunization (Nishiyama et al., 2009) displaying strong neutralizing activity in the PBMC assay did not impede pseudovirion entry into genetically engineered host cells expressing CD4 and chemokine receptors (TZM/Bl cells) (Paul et al., 2010). Antibodies to the CD4BD^OD^, in contrast neutralize the infection of TZM/Bl cells by pseudovirions. The reason for discrepant neutralizing activity remains to be elucidated, but a potential factor is overexpression of the HIV coreceptors resulting in decreased dependence of the infection process on the CD4BD^core^ residues essential for antibody recognition (Paul et al., 2010). Chemokine receptor saturation can result in artifactual inhibition of HIV infection. Certain antibodies and contaminant endotoxin can induce the host cells to release β-chemokines that inhibit infection of PBMCs (Geonnotti et al., 2010; Moody et al., 2010). However, neutralization by antibodies to the CD4BD^core^ occurs by recognition of HIV, not the host cells. For example, HIV neutralization increased progressively with increasing time of antibody contact with HIV while holding PBMC exposure to antibodies constant, ruling out anti-host cell effects (Planque et al., 2012b). Also, the antibody neutralizing activity was preserved in the presence of anti-β-chemokine antibodies. Additional evidence for genuine neutralization attributable to CD4BD^core^ recognition is available: the neutralizing activity was enriched in the CD4BD^core^-specific subset of polyclonal antibodies; CD4BD^core^-specific recombinant antibodies displayed neutralizing activity; the CD4BD^core^ peptide inhibited antibody neutralization competitively; neutralizing monoclonal antibodies were induced by immunization with E-gp120; and the antibodies recognized amino acids within the CD4BD^core^ important for CD4 binding (Planque et al., 2010). It may be concluded that the PBMC-based assay is a valid measure of specific CD4BD^core^ recognition and HIV neutralization.

## Conclusion

A candidate HIV vaccine directed to the CD4BD must address the problems of poor CD4BD immunogenicity and induction of antibodies that recognize the native CD4BD conformation. The CD4BD^OD^ and CD4BD^core^ epitopes present distinct immunogenicity and structural problems that are associated with epitope discontinuities, conformational instability, and B cell superantigenicity. Neutralizing antibodies to both types of epitopes are available. Currently the only immunogen demonstrated to induce a broadly neutralizing antibody response is electrophilic E-gp120, a protein that reacts covalently with nucleophilic BCRs. This immunogen addresses at least in part the problems of CD4BD superantigenicity and conformational flexibility. These problems are not limited to HIV. Other microbes such as *Staphylococcus aureus* express B cell superantigens. The principle of covalent immunization with electrophilic immunogens holds potential as a novel vaccine method that amplifies and improves the small set of innate antibodies capable of recognizing superantigens.

### Conflict of interest statement

All authors have a financial interest in patents covering the catalytic antibody and covalent vaccination area. Yasuhiro Nishiyama and Stephanie Planque are consultants for Covalent Bioscience Inc. and have a financial interest in the company.
